# Standardization of body composition parameters between GE Lunar iDXA and Hologic Horizon A and their clinical impact

**DOI:** 10.1093/jbmrpl/ziae088

**Published:** 2024-07-10

**Authors:** Colin Vendrami, Guillaume Gatineau, Elena Gonzalez Rodriguez, Olivier Lamy, Didier Hans, Enisa Shevroja

**Affiliations:** Rheumatology Unit, Bone and Joint Department, Interdisciplinary Center of Bone Diseases, Lausanne University Hospital and University of Lausanne, 1005 Lausanne, Vaud, Switzerland; Internal Medicine Unit, Medicine Department, Lausanne University Hospital and University of Lausanne, 1005 Lausanne, Vaud, Switzerland; Rheumatology Unit, Bone and Joint Department, Interdisciplinary Center of Bone Diseases, Lausanne University Hospital and University of Lausanne, 1005 Lausanne, Vaud, Switzerland; Rheumatology Unit, Bone and Joint Department, Interdisciplinary Center of Bone Diseases, Lausanne University Hospital and University of Lausanne, 1005 Lausanne, Vaud, Switzerland; Rheumatology Unit, Bone and Joint Department, Interdisciplinary Center of Bone Diseases, Lausanne University Hospital and University of Lausanne, 1005 Lausanne, Vaud, Switzerland; Internal Medicine Unit, Medicine Department, Lausanne University Hospital and University of Lausanne, 1005 Lausanne, Vaud, Switzerland; Rheumatology Unit, Bone and Joint Department, Interdisciplinary Center of Bone Diseases, Lausanne University Hospital and University of Lausanne, 1005 Lausanne, Vaud, Switzerland; Rheumatology Unit, Bone and Joint Department, Interdisciplinary Center of Bone Diseases, Lausanne University Hospital and University of Lausanne, 1005 Lausanne, Vaud, Switzerland

**Keywords:** body composition, DXA, standardization, calibration, Sarcopenia

## Abstract

Body composition (BC) measured by DXA differs between devices. We aimed to compare regional and total BC measurements assessed by the Hologic Horizon A and the GE Lunar iDXA devices; to determine device-specific calibration equations for each BC parameter; and to assess the impact of this standardization procedure on the assessment of sarcopenia, lipedema, obesity, and cardiovascular risk with DXA. A total of 926 postmenopausal women (aged 72.9 ± 6.9 yr, height 160.3 ± 6.6 cm, weight 66.1 ± 12.7 kg) underwent BC assessment on each device within 1 h, following the ISCD guidelines. The included sample was split into 80% train and 20% test datasets stratified by age, height, and weight. Inter-device differences in BC parameters were assessed with Bland–Altman analysis, Pearson or Spearman correlation coefficients, and *t*-tests or Wilcoxon tests. The equations were developed in the train dataset using backward stepwise multiple linear regressions and were evaluated in the test dataset with the R-squared and mean absolute error. We compared the abovementioned BC-derived health conditions before and after standardization in the test set with respect to relative risk, accuracy, Kappa score, and McNemar tests. Total and regional body masses were similar (*p*>.05) between devices. BMC was greater for all regions in the Lunar device (*p*<.05), while fat and lean masses differed among regions. Regression equations showed high performance metrics in both datasets. The BC assessment from Hologic classified 2.13 times more sarcopenic cases (McNemar: *p*<.001), 1.39 times more lipedema (*p*<.001), 0.40 times less high cardiovascular risk (*p*<.001), and similarly classified obesity (*p*>.05), compared to Lunar. After standardization, the differences disappeared (*p*>.05), and the classification metrics improved. This study discusses how hardware and software differences impact BC assessments. The provided standardization equations address these issues and improve the agreement between devices. Future studies and disease definitions should consider these differences.

## Graphical Abstract

**Graphical Abstract:**
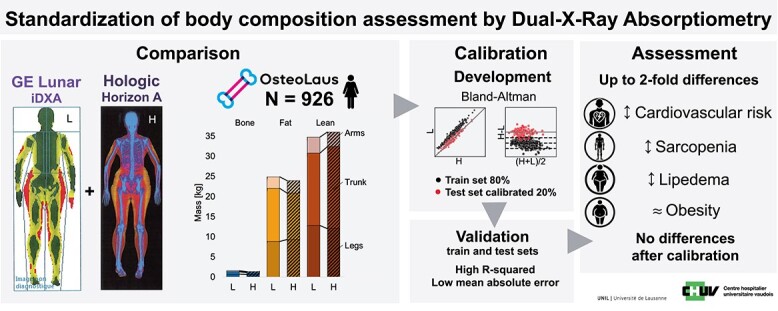

## Introduction

Body composition (BC) analysis using DXA is an accessible and versatile tool for assessing and monitoring fat, lean, and bone tissues. Since the first assessment of BC using body surface area and BMI, BC has been extensively described using modern methods, including anthropometric measurements and equations, chemical analyses, and imaging techniques.^1^^,^^2^ The current gold standards are magnetic resonance imaging for lean mass (LM),^3^ the 5-compartment model for fat mass (FM),^4^ and DXA for BMC.^5^ DXA is the most widely used tool because it is a fast, accurate, and low-radiation procedure with high reliability.^6^^,^^7^ DXA measures the differential absorption of 2 X-ray energies to estimate the amount and distribution of fat, lean, and bone tissues.^8^ Its use in research and clinical practice consists of the assessment of bone mass (osteoporosis, pediatric bone diseases), LM (sarcopenia, sports medicine), and FM (cardiovascular risk, obesity, lipedema and fat distribution, pharmacology).^9–15^

Previous studies have shown a variability of approximately 1.0% for most DXA parameters, ranging from 0.2% for total mass to 15.9% for visceral FM.^15–17^ A literature search in PubMed identified 18 articles comparing the use of DXA devices based on their manufacturer and models (see List 1).^18–35^ None of these studies compared the 2 latest generations of DXA devices, and only one assessed the difference in the classification of BC-derived health conditions.^19^

The objectives of this study were (1) to compare the BC parameters assessed by the Hologic Horizon A System™ and GE Lunar iDXA™ on the OsteoLaus cohort and on an anthropomorphic whole-body phantom, (2) to provide calibration equations for each BC parameter between both devices, and (3) to assess the impact of this standardization procedure on the classification of BC parameter-derived health conditions.

## Materials and methods

### Study population

This study followed the STROBE checklist for prediction model development and validation (Table S1). The OsteoLaus cohort is a population-based study (Lausanne, Switzerland) and 10-yr prospective study aiming to analyze fragility fractures.^36^ OsteoLaus is a substudy of the CoLaus|PsyColaus study, an ongoing prospective study initiated in 2003 to assess the determinants of cardiovascular and psychiatric diseases in a population-based sample.^37^^,^^38^ Between September 2009 and September 2012, all women aged 50–80 yr from the CoLaus|PsyColaus study were invited to participate in the OsteoLaus study. Of the initial 1704 women invited, 1500 agreed to participate and 1475 were included; 98.4% were Caucasian. OsteoLaus was followed up every 2.5 yr for 10 yr, first in 2012, second in 2015, third in 2018, and fourth in 2020. At each visit, the patients completed a questionnaire on previous fragility fractures, medications, and osteoporosis risk factors; they underwent physical examinations (anthropometry, muscle testing, heel ultrasound), bone health, and BC assessment by DXA (see next section). This study used the fourth OsteoLaus follow-up, involving 944 women followed from March 2020 to October 2022, during which each participant underwent BC assessment with 2 different DXA devices on the same day within 1 h of each other.

**List 1:** Studies comparing total body composition assessment by DXA.

First author, year ^ref.^	Population	Origin	Age (years)	Devices	Cross-calibration equations
**Maïmoum, 2023^18^**	9 males 46 females	France	41 ± 13.0	DMS Stratos Hologic QDR 4500A	Yes
**Bennett, 2023^19^**	17 males 97 females	China	51.1 ± 16.5	Lunar Prodigy Hologic Discovery/A	Yes
**Lyons-Reid, 2022^20^**	28 children	New Zealand	3.4 ± 0.2	GE Lunar iDXA GE Lunar Prodigy	Yes
**Ha, 2021^21^**	26 males 14 females	Korea	24.9 ± 1.69	GE Lunar Prodigy Osteosys Primus	Yes
**Reitshamer, 2021^22^**	21 males 23 females	USA	72 ± 9.8	GE Lunar Prodigy Hologic Horizon A	Yes
**Park, 2021^23^**	20 males 20 females	Korea	42.8 ± 18.8	GE Lunar Prodigy Hologic Horizon W	Yes
**Khadilkar, 2019^24^**	126 children 135 adults	India	10.7 ± 5.0 35.6 ± 12.2	GE Lunar iDXA GE Lunar DPX-Pro	Yes
**Oldroyd, 2018^25^**	28 males 55 females	UK	45.3 ± 12.8	GE Lunar iDXA GE Lunar Prodigy	Yes
**Watson, 2017^26^**	33 males 36 females	UK	37 ± 12	GE Lunar iDXA GE Lunar Prodigy	Yes
**Xu, 2016^27^**	36 males 60 females	USA	36.2 ± 14.0	GE Lunar iDXA Hologic Discovery	No
**Ito, 2015^28^**	15 males 15 females	Japan	35.0 ± 10.0	GE Lunar DPX-NT Hologic QDR-4500 C	Yes
**Shepherd, 2012^29^**	21 males/ 19 females 40 males/ 119 females	USA China	36.8 ± 23.2	GE Lunar Prodigy Hologic Discovery or Delphi	Yes
**Pearson, 2011^30^**	2 males/ 19 females	UK	55 (30-79)	GE Lunar Prodigy Hologic QDR-2000	Yes
**Covey, 2010^31^**	38 females	USA	50.6 ± 9.6	Hologic Discovery Wi Hologic QDR-4500 W	No
**Hull, 2009^32^**	47 males 52 females	USA	46.3 ± 16.9	GE Lunar iDXA GE Lunar Prodigy GE Lunar DPX	Yes
**Ellis, 2009^33^**	33 children 14 adults	USA	NA	Hologic QDR-4500 A Hologic QDR-2000 W	Yes
**Aasen, 2006^34^**	8 males 13 females	Norway	61.5 ± 12.5	GE Lunar Prodigy GE Lunar Expert Hologic Delphi W	Yes
**Soriano, 2004^35^**	39 males 39 females	USA	From 19 to 81	Hologic Delphi A GE Lunar Prodigy GE Lunar DPX GE Lunar DPX-L	Yes

Publications retrieved on PubMed using a simple search syntax and no time restriction.

### DXA

#### DXA devices features

The GE Lunar iDXA (L-iDXA) system is a dual-energy X-ray narrow fan beam. The perpendicular X-ray beam emerging from under the table follows a square wave to scan from the patient’s head to the feet. The total body scan time ranged from 7 min 16 s to 13 min 16 s, depending on the patient’s morphology. The X-ray dose varies accordingly from 3 to 6 μGrays. The device’s maximum patient height is 198 cm, the width is 66 cm, and the weight is 204 kg. The image was then analyzed using enCORE v16.0 (GE Healthcare), which includes CoreScan for the assessment of visceral adipose tissue (VAT).^39^

The Hologic Horizon A System (H-Horizon-A) is a dual-energy X-ray fan beam with k-edge filtering (3 filters: 6 energies in total). The X-ray beam emerging from the C-arm follows 3 movements (1 pass at side with 30° beam angle, 1 pass at the center perpendicularly, and 1 last pass on the other side at 30°) for a total duration of 2 min 54 s and an X-ray dose of 7 μGrays. The device maximum patient height is 195.6 cm, the width is 67 cm, and the weight is 227 kg. The image was reconstructed and analyzed using Hologic Apex software V5.6.1.3 (Hologic).^40^

A whole body phantom (NO 1207, Hologic®) was scanned 10 times on both devices without repositioning. The phantom contains a known amount of BC tissues: 6 layers of high-density polyethylene and polyvinyl chloride mimicking fat and lean tissues, respectively. Geometric aluminum shapes are embedded to simulate bone tissues. The total weight of the phantom is 28 kg, and the length, width, and height of the phantom are 76 × 36 × 14 cm. No cross-calibration was undertaken between the devices at the beginning of the study, and the devices are thus expected to represent their default configuration.

#### DXA scan acquisition

Scans were performed by 2 technicians according to the ISCD guidelines.^12^ None of the participants had undergone contrast imaging in the previous 3 wk. All participants wore medical coats, and all metals and jewelry were removed when possible. For both DXA scans, participants lay supine in the center of the scan field with their arms at their sides, separated from the trunk. The hand palms were facing down for Hologic and to the side for Lunar. The ankles were strapped together. If the positioning was inaccurate and/or the participant moved during the scan acquisition, the procedure was restarted. The ROIs were defined automatically by the respective DXA software and corrected manually by the technician if the ROI did not follow the International Society for Clinical Densitometry (ISCD) guidelines.^12^ Region mirroring (or “reflection”) was used when the scanning field was too narrow for the patient’s morphology. Secondary visual and qualitative control was performed on each image: body parts with visible artifacts were excluded or replaced with region mirroring if possible.

#### DXA BC analysis

The variables of interest were derived from bone (BMC), lean (LM), and fat tissue (FM) for the total body and subregions (arms, legs, trunk, android, and gynoid). The subtotal body represents the total body without the head. The appendicular lean mass (ALM) is the sum of the LM of both arms and both legs. ALM divided by height squared (ALMI), or BMI (ALM/BMI), was also calculated. Percent fat (%FAT) is the total FM divided by total mass. The android region represents the lower part of the abdomen: the lower limit is the pelvic horizontal cut line, and the upper limit is placed above 20% of the distance between the pelvis and chin. The gynoid region represents the upper part of the thighs below the buttocks: the upper limit is located one and a half times the width of the android region below the pelvic cut line, and the width of the gynoid region is twice the width of the android region. Based on user manuals, VAT is derived from the android region for L-iDXA and from a smaller region within the android for Hologic. The exact calculation of VAT has not been fully described.^39^^,^^40^

BC analysis for Hologic was performed using the Classic or the NHANES Hologic BC calibration. The NHANES calibration setting is a postprocessing calibration that converts 5.4% of LM to FM, leaving bone unchanged.^41^^,^^42^ Lunar iDXA has only one default calibration.

#### Anthropometric measures

Height was measured with a portable stadiometer (Seca version 216, Seca) with a precision of 0.1 cm, and body weight was measured with an electronic scale (Seca Clara 803, Seca) with a precision of 0.1 kg, with the participant barefoot and dressed in a medical coat. BMI was calculated by dividing the participant’s weight by the square of their height (kg/m^2^).

### Statistical analysis

Statistical analysis and data visualization were performed in Python (v3.10.13) using the pandas (v2.1.4), seaborn (v0.12.2), statsmodels (v0.14.0), scypi.stats (v1.11.4), and scikit-learn (v1.3.0) libraries. The full Python functions are available upon request. To assess internal validity, we previously split the data into train (80%) and test (20%) datasets using stratification by age, weight, and height.

#### Between-devices comparison

Each pair of variables was described by the mean for each DXA device and the absolute and relative differences between the means. For the statistical comparison of normally distributed variables, paired sample-dependent or independent *t*-tests were used. A *p*-value lower than .05, corresponding to a 95% CI, was considered significant. When a variable was not normally distributed, based on the Shapiro–Wilk test, violin plots, and quantile–quantile plots, statistical comparisons were performed using the Wilcoxon Mann–Whitney U test. The correlation between the variables of the 2 instruments was assessed using the Pearson or Spearman coefficient, depending on the distribution. All these previous steps were performed similarly for the whole body phantom. A Bland–Altman analysis was used to visually describe the agreement of the paired values (X and Y), including 3 graphs: (1) a scatter plot of the raw X and Y values, (2) the constant agreement based on their mean (X + Y)/2 and their difference (Y−X), and (3) the relative agreement based on their mean (X + Y)/2 and their relative difference Y−X/((X + Y)/2).^43^ These graphs include both the train and the calibrated test datasets (see the next section).

#### Development and validation of the calibration equations

The derivation of the calibration equations followed an iterative approach using multivariate and univariate models for each parameter in the train dataset. We included fat, lean, and bone as covariates based on their known associations in the 3-compartment analysis.^5^ We first included all 3 tissues in a backward stepwise multivariate linear regression for each variable separately. If the only covariate with a significant coefficient (*p*<.05) was a measure similar to that of the other device, we then performed a univariate regression using Deming regression.^44^ If the previous multivariate model had more than one variable with a significant coefficient (*p*<.05), we performed 2 additional steps to choose between the multivariate or univariate model. First, the pattern of association of each included covariate was visually assessed using continuous color maps and percentile-based subgroup analysis (quintiles, tertiles and mean height, weight, and total body FM), and the importance of each covariate was visualized using bar charts Figures S3.1–S3.2 as example). Second, for both the uni- and multivariate models, their performances were assessed in the train and test datasets by comparing the difference between one device and the other after calibration using: the mean difference, paired *t*-tests, R-squared (R^2^), and the mean absolute error (MAE). To simplify these supplementary tables, the equations for both devices are shown, but only the evaluation metrics from Lunar to Hologic are shown (Tables S2A–C). The evaluation metrics using the inverse models from Hologic to Lunar were not different. All evaluation metrics were used for both model selection and the model internal validation.

#### Between-device differences in BC-derived health conditions and the impact of standardization

As a complementary analysis, we examined the classification of different clinical conditions in the independent test dataset, such as low LM in sarcopenia,^11^ a high percentage of body fat in obesity,^9^ high VAT in cardiovascular risk assessment,^10^ and high leg FM over total FM in lipedema.^14^ We evaluated the differences in proportions using the McNemar test, the classification performance using the Cohen kappa coefficient, and the accuracy. These steps were performed before and after standardization with the developed calibration equations.

## Results

### Participants characteristics

**Figure 1:**
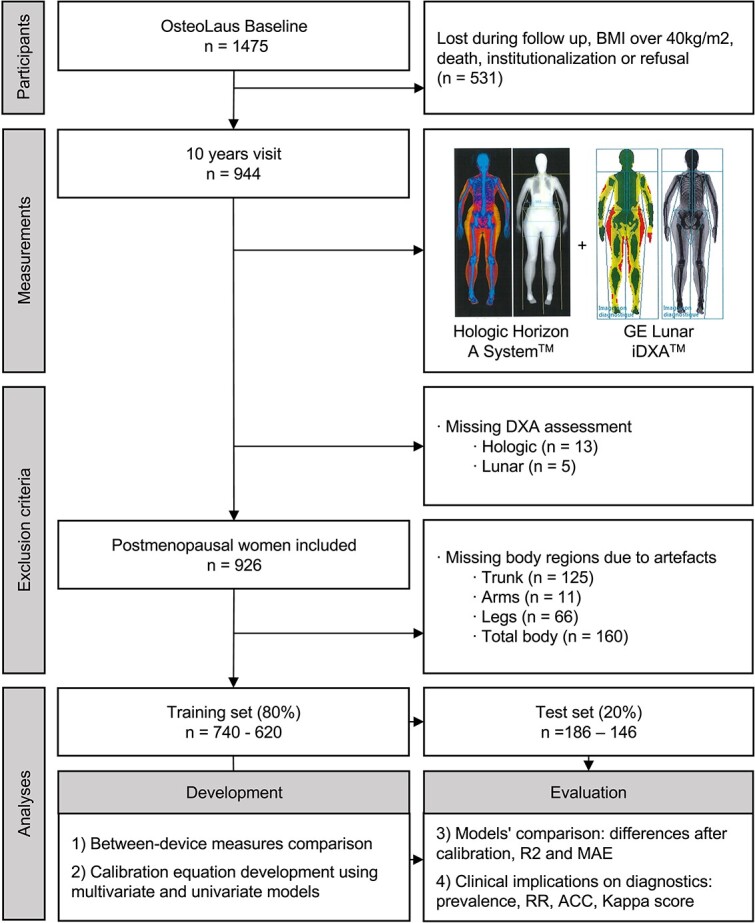
Flowchart of data collection and analysis. *Legend*: the type of artefacts are described in Figure S1. *Abbreviations*: *n*, number; R^2^, R-squared; MAE, mean absolute error; RR, relative risk; ACC, accuracy; Kappa score, Cohen kappa score.

Of the initial 944 postmenopausal women who attended the last OsteoLaus visit, 926 underwent BC scanning with both devices (Figure 1. Study flowchart). The mean age was 72.9 ± 6.9 yr, height was 160.3 ± 6.6 cm, weight was 66.1 ± 12.7 kg, and BMI was 25.7 ± 4.8 kg/m^2^ (Table 1). These characteristics were not significantly different between the included and excluded participants or between the participants in the train (*n* = 740, 80%) and test datasets (*n* = 186, 20%). For each regional and total body analysis, 308 participants had 391 DXA artifacts. These artifacts were corrected with mirror imaging when possible or were excluded otherwise: the remaining noncorrected artifacts were in the trunk (*n* = 125), arms (*n* = 11), legs (*n* = 66), or total body (*n* = 160) (Figure 1 and Figure S1). The final sample size in each subanalysis varied accordingly. The detailed sample sizes, including artifact exclusion, are shown in Table 2 and Tables 3A and 3B, and Table S2.1C.

**Table 1:** Included and excluded participants’ characteristics.

	Included, *n* = 926			Excluded, *n* = 18	
	Training set	Test set	*p*-value		*p*-value
*n*	740 (80%)	186 (20%)			
**Age (yr)**	72.9 ± 6.8 (60.1–91.5)	72.9 ± 7.1 (60.4–90.2)	.81	74.9 ± 8.8 (62.4–87.7)	.26
**Weight (kg)**	66.1 ± 12.7 (36.3–114.3)	66.1 ± 13.1 (36.3–110.0)	.98	69.4 ± 8.7 (54.2–88.1)	.14
**Height (cm)**	160.4 ± 6.5 (138.7–180.4)	160.2 ± 7.0 (137.9–180.2)	.79	161.7 ± 7.0 (151.6–172.5)	.41
**BMI (kg/cm^2^)**	25.7 ± 4.8 (16.6–45.5)	25.8 ± 4.9 (16.6–40.7)	.94	26.7 ± 4.0 (21.2–33.4)	.27

Eighteen participants excluded for missing DXA were compared to the 926 included. The included participants were split (80% training—20% test) using a stratification for age, weight, and height, and compared. Results are shown as mean ± standard deviation (min-max). Mann–Whitney U test was used to compare non-normally distributed variables (age, weight, BMI) and independent *t*-test for normally distributed variable (height).

Abbreviations: *n*, number; yr, year; kg, kilograms; cm, centimeters.

**Table 2:** Area and mass: comparison of total body and regional measures, absolute, and relative differences.

		Train (*n*)	Lunar	Hologic	Absolute difference	*p* ^a^	Relative difference	Correlation^b^
**Total mass (g)**	Total body	620	65 379.4 ± 12 155.0	65 601.8 ± 12 264.4	222.4 ± 383.2	.72	< 0.01 ± 0.01	0.999
	Weight by scale	620	66 088.0 ± 12 651.6		L—scale: −139.2 ± 491.7 H—scale: 83.3 ± 539.1	L: 0.39 H: 0.63	L: < −0.01 ± 0.01 H: < 0.01 ± 0.01	L: 0.999 H: 0.999
	Subtotal body	620	61 078.4 ± 11 920.0	61 334.5 ± 12 022.1	256.1 ± 505.9	.68	<0.01 ± 0.01	0.999
	Arms	734	7093.9 ± 1588.9	7139.5 ± 1526.2	45.7 ± 416.2	.37	0.01 ± 0.05	0.970
	Legs	688	22 448.0 ± 4608.9	22 074.6 ± 4488.0	−373.4 ± 664.5	.14	−0.02 ± 0.03	0.988
	Trunk	646	31 772.3 ± 6539.6	32 387.0 ± 6902.8	614.6 ± 850.3	.13	0.02 ± 0.03	0.994
**Area (cm^2^)**	Total body	740	1933.3 ± 152.7	1815.6 ± 155.0	−117.7 ± 111.1	<.001^c^	−0.06 ± 0.06	0.739^c^
	Subtotal body	740	1709.7 ± 149.0	1601.1 ± 150.3	−108.6 ± 111.3	<.001^c^	−0.07 ± 0.07	0.723^c^
	Arms	740	335.3 ± 32.3	384.9 ± 43.4	49.6 ± 39.0	<.001	0.14 ± 0.10	0.493
	Legs	740	711.8 ± 66.4	664.7 ± 70.0	−47.1 ± 51.5	<.001	−0.07 ± 0.08	0.736
	Trunk	740	662.6 ± 75.3	501.1 ± 59.1	−161.5 ± 62.9	<.001	−0.28 ± 0.11	0.597

*n* = number of participant finally included after exclusion (cf. Figure 1); Lunar (L), Hologic (H), and absolute difference (H-L) are expressed as mean +/− standard deviation in grams (gr.)

a *p*-value from dependent *t*-test^c^ or Mann–Whitney U test (default); relative difference = H−L/((H + L)/2).

b Correlation coefficient with Pearson^c^ or Spearman correlation (default).

**Table 3:** (A) Fat mass: comparison of total body and regional measures, absolute and relative differences, and proposed calibration equations.

Tissue and region		*n*		Train set							R^2^	
		Train test		Lunar	Hologic	Absolute difference	*p* ^a^	Relative difference	Corr-elation^b^	Equations^c^	Train test	
**Fat mass (g)**	Total body	620–146		25 602.8 ± 8921.7	24 800.4 ± 7786.6	−802.4±1501.2	.19	−0.02±0.06	0.993	H = 2476.1 + 0.872*L_WBTOT_FAT L = −2839.7 + 1.147*H_WBTOT_FAT	H: 0.986 0.983 L: 0.986 0.984	
	Total body percent (%)	620–146		38.1±7.1	37.0±5.7	−1.1±2.1	.001	−0.02±0.06	0.968	H = 6.7 + 0.795*L_WBTOT_PFAT L = −8.4 + 1.258*H_WBTOT_PFAT	H: 0.942 0.921 L: 0.941 0.924	
	Subtotal body	620–146		24 742.7 ± 8847.0	23 766.8 ± 7723.0	−975.9±1499.0	.09	−0.03±0.06	0.993	H = 2188.7 + 0.872*L_SUBTOT_FAT L = −2509.6 + 1.147*H_SUBTOT_FAT	H: 0.986 0.982 L: 0.986 0.983	
	Arms	734–181		2870.7 ± 1090.7	3110.4±1151.4	239.7±346.9	<.001	0.08±0.10	0.955	H = 72.0 + 1.058*L_ARMS_FAT L = −68.0 + 0.945*H_ARMS_FAT	H: 0.907 0.921 L: 0.907 0.919	
	Legs	688–172		8867.9 ± 3052.5	9350.8±2976.4	482.9±463.1	.001	0.06±0.06	0.987	H = 706.5 + 0.975*L_LEGS_FAT L = −724.8 + 1.026*H_LEGS_FAT	H: 0.977 0.979 L: 0.977 0.979	
	Trunk	646–155		13 188.5 ± 5440.0	11 534.5 ± 4420.8	−1654.1±1298.9	<.001	−0.11±0.09	0.989	H = 846.8 + 0.810*L_TRUNK_FAT L = −1044.9 + 1.234*H_TRUNK_FAT	H: 0.973 0.975 L: 0.973 0.976	
	Android	646–155		2175.1 ± 1065.8	2168.5±923.3	−6.6±237.2	.74	0.04±0.14	0.985	H = 289.3 + 0.864*L_ANDROID_FAT L = −334.9 + 1.157*H_ANDROID_FAT	H: 0.964 0.962 L: 0.963 0.963	
	Gynoid	646–155		4329.5 ± 1438.3	4650.5±1308.2	321.0±338.9	<.001	0.09±0.09	0.969	H = 744.8 + 0.901*L_GYNOID_FAT L = −827.0 + 1.110*H_GYNOID_FAT	H: 0.950 0.939 L: 0.950 0.941	
	VAT	646–155		842.8±594.6	560.2±263.8	−282.5±370.0	<.001	−0.23±0.40	0.938	H = 209.1 + 0.417*L_VAT_MASS L = −502.0 + 2.400*H_VAT_MASS	H: 0.832 0.852 L: 0.809 0.805	

*n* = number of participant finally included after exclusion and data splitting process (cf. Figure 1); Lunar (L), Hologic (H), and absolute difference (H-L) are expressed as mean +/− standard deviation in grams (gr.).

Abbreviations, WBTOT, whole body total; SUBTOT, subtotal body; PFAT, percent fat = total fat mass / total mass; VAT, visceral adipose tissue

a *p*-value from Mann–Whitney U test; relative difference = H−L/((H + L)/2).

b Spearman rank correlation coefficient.

c Equations (see Table S.2. A for the model selection): H, Hologic Horizon A^Classic setting^; L, Lunar iDXA; R^2^, R-squared = proportion of variance explained by the model.

**Table 3:** (B) Lean mass: comparison of total body and regional measures, absolute and relative differences, and proposed calibration equations.

Tissue and region		*n*		Train set							R^2^	
		Train test		Lunar	Hologic	Absolute difference	*p* ^a^	Relative difference	Correlation^b^	Equations^c^	Train test	
**Lean mass (g)**	Total body	620–146		37 703.7 ± 4459.4	38 997.5 ± 5356.2	1293.7 ± 1643.5	<.001	0.03±0.04	0.953	H = −6628.8 + 1.210*L_WBTOT_LEAN L = 5477.8 + 0.826*H_WBTOT_LEAN	H: 0.920 0.912 L: 0.921 0.906	
	Subtotal body	620–146		34 738.5 ± 4315.0	36 207.0 ± 5186.2	1468.5 ± 1628.9	<.001	0.04±0.04	0.949	H = −5882.1 + 1.212*L_SUBTOT_LEAN L = 4854.9 + 0.825*H_SUBTOT_LEAN	H: 0.915 0.908 L: 0.916 0.901	
	Arms	734–181		3961.5 ± 633.1	3810.1 ± 608.3	−151.4±274.8	<.001	−0.04±0.07	0.898	H = 19.8 + 0.957*L_ARMS_LEAN L = −20.6 + 1.045*H_ARMS_LEAN	H: 0.806 0.818 L: 0.805 0.802	
	Legs	688–172		12 830.9 ± 1977.0	12 053.8 ± 1955.5	−777.1±490.0	<.001	−0.06±0.04	0.966	H = −633.8 + 0.989*L_LEGS_LEAN L = 640.9 + 1.011*H_LEGS_LEAN	H: 0.938 0.936 L: 0.938 0.934	
	Trunk	646–155		17 999.6 ± 2068.2	20 412.3 ± 2957.2	2412.6 ± 1628.9	<.001	0.12±0.08	0.822	H = −418.0 + 0.957*L_TRUNK_LEAN + 0.273*L_TRUNK_FAT L = 3379.4 + 0.865*H_TRUNK_LEAN −0.242*H_TRUNK_FAT −0.436*L_TRUNK_BMC	H: 0.940 0.934 L: 0.848 0.818	
	ALM	683–167		16 770.7 ± 2515.9	15 853.5 ± 2436.0	−917.2±594.3	<.001	−0.06±0.04	0.967	H = −369.3 + 0.967*L_ALM L = 381.8 + 1.034*H_ALM	H: 0.943 0.939 L: 0.943 0.935	

*n* = number of participant finally included after exclusion and data splitting process (cf. Figure 1); Lunar (L), Hologic (H), and absolute difference (H-L) are expressed as mean +/− standard deviation in grams..

Abbreviations: WBTOT, whole body total; SUBTOT, subtotal body; ALM, appendicular lean mass.

a *p*-value from Mann–Whitney U test; relative difference = H−L/((H + L)/2).

b Spearman rank correlation coefficient.

c Equations (see Table S.2. B for the model selection): H: Hologic Horizon A^Classic setting^; L: Lunar iDXA; R^2^: R-squared = proportion of variance explained by the model.

### BC parameters: Between-device comparison

**Figure 2:**
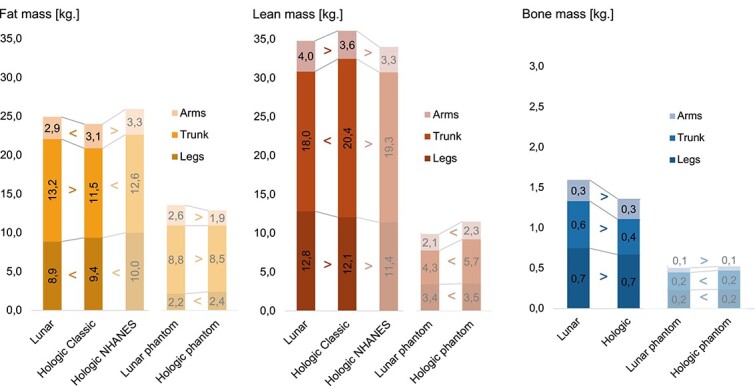
Comparison of fat, lean, and bone mass mean values measured with Lunar iDXA and Hologic Horizon (Classic and NHANES settings) devices in postmenopausal women and in a Hologic whole body phantom. *Legend*: comparison of the mean fat, lean, and bone mass (kg) values. For each regions, all means were statistically different between devices/settings (*p*<.005) using Mann–Whitney U Test.

A comprehensive summary of all values, including those of the OsteoLaus participants and the whole-body phantom, is presented in Figure 2. The values for total mass and area for both devices are compared numerically in Table 2. The values for FM, LM, and BMC are compared in Tables 3A and 3B, and Table S2.1C, respectively. A visual comparison with a Bland–Altman analysis is available in Figures S2A and B, and Table S2.2C.

#### Mass and area

Comparisons of total mass measured with L-iDXA and H-Horizon-A revealed no significant differences between the devices or between the DXA devices and the electronic scale (Table 2). In terms of area, L-iDXA measured larger area than H-Horizon-A for the total body (Table 2).

#### Fat mass

Total FM was not significantly different between devices: the FM of L-iDXA was 802.3 g (+2.0%, *p*=.19) greater than that of H-Horizon-A (Table 3A and Figure S2A). The percentage of FM (%FAT) was 1.1% (+2.0%, *p*=.001) greater for L-iDXA than for H-Horizon-A. VAT was 282.5 g (+22.9%, *p*<.001) greater for L-iDXA than for H-Horizon-A.

#### Lean mass

The total LM was 1293.7 g (−3.1%, *p*<.001) lower for L-iDXA than for H-Horizon-A (Table 3B and Figure S2B). The trunk LM was 2412.6 g (−12.2%, *p*<.001) lower for L-iDXA, while the masses of the arms and legs were 151.4 g (+3.9%, *p*<.001) and 777.1 g (+6.4%, *p*<.001) greater for L-iDXA, respectively.

#### Bone mineral content

The total BMC was 268.9 g greater (+14.0%, *p*<.001) for L-iDXA than for H-Horizon-A (Table S2.1Cand Figure S2C). The subtotal BMD was 0.076 g/cm^2^ (+8.3%, *p*<.001) greater for L-iDXA than for H-Horizon-A.

#### Alternative Hologic calibration settings

L-iDXA values were compared to those of the H-Horizon-A Classic or NHANES calibration (Figure 2 and Table S3). Overall, the H-Horizon-A NHANES calibration systematically yielded greater FM values and lower LM values than did the H-Horizon Classic calibration (*p*<.05).

#### Whole-body phantom

In a second parallel analysis using a Hologic whole-body phantom, total LM and total BMC were lower for L-iDXA than for H-Horizon-A, while total FM was greater for L-iDXA (*p*<.001, Figure 2 and Table S4.).

### Calibration equations development and validation

The final and selected calibration equations developed on the train dataset are summarized on the right side of Tables 3A–C and S2.1C. All univariate and multivariate models are available in Tables S2A and B, and S2.2C). The proportion of variance explained by the univariate models (R-squared) ranged from 0.672 for trunk LM to 0.986 for total body FM. According to the multivariate models, this proportion ranged from 0.803 for trunk BMC to 0.989 for total body FM. The trunk was also the region with the greatest improvement between the performance metrics (R^2^, MAE, and difference of means after calibration) when comparing the multivariate and univariate models (Tables S2A–C). All equations tested in the independent test dataset showed similar R^2^ and MAE values to those of the development test dataset. After calibration in the test dataset, all means for all variables were not significantly different (*p*>.05) between the calibrated value and the value used as a reference (delta^c^).

In the complementary analysis, the most divergent results were for trunk LM and trunk BMC, with different linear equations for each percentile-based group (Figure S3.1). The interaction of the covariates was visible in the color gradient and in the bar plot (Figure S3.1). ALM showed fewer interaction using the similar analysis (Figure S3.2).

### Between-device differences in BC-derived health conditions and the impact of device standardization

Based on the 167 participants from the test dataset (Table 4), sarcopenia, as defined for individuals with an ALM less than 16 kg, was 1.37-fold greater for H-Horizon-A than for L-iDXA (McNemar: *p*<.001). Sarcopenia based on an ALMI less than 5.5 kg/m^2^ was also 2.13-fold greater for H-Horizon-A than for L-iDXA (*p*<.001). The relative risk (RR) of obesity, as described by a total %FAT above 35%, was 1.06-fold greater for H-Horizon-A than for L-iDXA (*p*>.05). The RR of high cardiovascular risk based on VAT above 839 g was 2.5-fold greater for L-iDXA than for H-Horizon-A (*p*<.001). The RR of lipedema, defined as leg FM/total FM above 0.383, was 1.38-fold greater for H-Horizon-A than for L-iDXA (*p*<.001). Applying calibration equations to BC parameters reduced the relative risk, improved the accuracy, and improved the level of agreement (kappa score) between both devices in the classification of these health conditions. Similarly, the differences in prevalence (McNemar) between the devices after calibration became nonsignificant (*p*>.05) (Table 4).

**Table 4:** Prevalence of clinical diagnosis cut-offs per devices before and after cross-calibration in the test dataset.

Disease (Assessment^ref^)	Cut-offs	Number of participants (% of test dataset, *n* = 167)				Relative risk	Kappa coef.	Accuracy	*p*-value
		Lunar	Hologic	Hologic^c^	Lunar^c^				
**Sarcopenia** **(low ALM^11^)**	ALM < 16 kg	67 (40.1%)	92 (55.1%)	65 (38.9%)	94 (56.3%)	L-H: 1.37 L-H^c^: 0.97 L^c^-H: 0.98	L-H: 0.71 L-H^c^: 0.85 L^c^-H: 0.88	L-H: 0.85 L-H^c^: 0.93 L^c^-H: 0.94	L-H: <.001 L-H^c^: .77 L^c^-H: .75
	ALMI < 5.5 kg/m^2^	16 (9.6%)	34 (20.4%)	19 (11.4%)	37 (22.2%)	L-H: 2.13 L-H^c^: 1.19 L^c^-H: 1.09	L-H: 0.59 L-H^c^: 0.71 L^c^-H: 0.77	L-H: 0.89 L-H^c^: 0.95 L^c^-H: 0.92	L-H: <.001 L-H^c^: .51 L^c^-H: .58
**Obesity** **(high percent fat^9^)**	Total %FAT > 35%	48 (32.9%)	51 (34.9%)	47 (32.2%)	51 (34.9%)	L-H: 1.06 L-H^c^: 0.98 L^c^-H: 1.00	L-H: 0.86 L-H^c^: 0.83 L^c^-H: 0.85	L-H: 0.94 L-H^c^: 0.93 L^c^-H: 0.93	L-H: .51 L-H^c^: 1.00 L^c^-H: 1.00
**Cardiovascular risk** **(high visceral fat^10^)**	VAT > 839 g	67 (43.2%)	27 (17.4%)	67 (43.2%)	23 (14.8%)	L-H: 0.40 L-H^c^: 1.00 L^c^-H: 1.17	L-H: 0.43 L-H^c^: 0.87 L^c^-H: 0.76	L-H: 0.74 L-H^c^: 0.94 L^c^-H: 0.94	L-H: <.001 L-H^c^: 1.00 L^c^-H: .34
**Lipedema** **(fat mass distribution^14^)**	Leg fat/total fat mass > 0.383 g/g	49 (33.6%)	68 (46.6%)	50 (34.2%)	69 (47.3%)	L-H: 1.39 L-H^c^: 1.02 L^c^-H: 0.99	L-H: 0.73 L-H^c^: 0.89 L^c^-H: 0.90	L-H: 0.87 L-H^c^: 0.95 L^c^-H: 0.95	L-H: <.001 L-H^c^: 1.00 L^c^-H: 1.00

All these cut-offs cannot diagnose the associated disease/health condition on their own and a broader clinical evaluation is necessary. Relative risk calculated from the proportion of each disease in H-Horizon-A vs L-iDXA; Kappa coef.: Cohen Kappa coefficient representing the level of agreement between 2 class; the accuracy is the fraction of identically classified participants; *p*-value from McNemar test.

*Abbreviations*: Hologic^c^, Hologic values calibrated toward Lunar using the equations; Lunar^c^, Lunar values calibrated toward Hologic using the equations; ALM, appendicular lean mass; ALMI, ALM/height^2^; %FAT: percent fat = (total fat mass/total mass) × 100; VAT, visceral adipose tissue.

## Discussion

### BC parameters assessed by both DXA devices

In summary, BMC assessments were greater for all regions in L-iDXA vs H-Horizon-A. The total body and trunk assessments were both significantly greater for FM and lower for LM in L-iDXA vs H-Horizon-A, while arms and legs assessment showed opposite results, with significantly lower fat and greater LM in L-iDXA vs H-Horizon-A. Eighteen other studies have shown how DXA devices differ in terms of bone, fat, and LM (see List 1: sample size: 21–199 individuals, mean age from 3.4 to 72 yr). A trend toward greater total fat for Lunar devices and greater total lean for Hologic devices seems consistent across studies, while regional comparisons, such as those of appendicular and trunk measures, have not been consistent.^18–35^ Interestingly, although the trunk was the region with the greatest difference, android FM was identical between the devices in our study. As the android region is a subcompartment of the trunk region (middle part of the trunk), the difference in FM observed in the trunk may thus be due to the upper (eg, thorax and upper abdomen) and/or lower part of the trunk (pelvis). Recently, another study also revealed a significant difference in the measurement of VAT between the Lunar Prodigy system and the Hologic Delphi system.^19^ Similarly, the VAT was the region with the greatest difference between the devices, with a 1.5-fold difference. This difference is probably mostly due to the difference in the region of interest, which is smaller in H-Horizon-A than in L-iDXA (see methodology).^19^

#### Hologic calibration setting

In 2005, Hologic BC analysis was based on an NHANES calibration.^42^ In 2020, Hologic reverted to classic calibration based on 2 studies comparing DXA with the 4-compartment model.^41^ Using the relative difference, the classic calibration results in fewer differences overall. Hence, we recommend following the manufacturer’s instructions using classic and uncalibrated analysis. We emphasize the importance of reporting which of the settings are being used (available directly on the report or in the DXA software settings).

#### Whole-body phantom and subgroup analyses

The whole-body phantom comparison showed similar overall differences between the devices, as for the OsteoLaus participants. As a phantom is smaller and lighter than a human, the size and weight of the scanned body/human do not appear to affect the overall measurement differences. In the regional comparison of the phantom (arms, trunk, legs), the differences were not comparable to those of the OsteoLaus participants.

### Standardization of BC parameters through calibration equations

All differences between the devices could be calibrated using specific equations developed within the train dataset. The differences in the evaluation metrics between the train and test datasets were small; therefore, the results are not expected to be internally overfitted. Overall, the final multivariate regression equations performed better than did the univariate equations in the train and test datasets (Tables S2A and B, and S2.2C). However, most of these improvements were of minimal clinical significance, and their inclusion could eventually lead to overfitting in external cohorts. We propose to use the multivariate cross-calibration equation only for trunk LM (Table 2B and Table S2B) and trunk BMC (Table S2.1, Figure S3.1).

In the subanalyses, the stratification of the OsteoLaus participants by weight or height resulted in relatively similar equations for total fat, total lean, and total BMC masses. The percentile-based analyses and the multivariate regressions showed few interplay of the different tissues except for the trunk LMs and trunk BMC measured by each device (Figures S3.1 and S3.2). Therefore, the equations are expected to hold in the case of follow-up with a change in BC (eg, ageing, change in health status, treatment).

FM appears to be an important covariable to consider in these comparisons. Similarly, Knapp et al. described greater variations in total body BMC in participants with higher BMI.^45^ Using the same device, this variation might be explained by beam hardening: lower X-ray energies are more likely to be attenuated by lower atomic number and density, as in nonbone tissues.^46^ To counteract part of this phenomenon, the acquisition time of L-iDXA is adapted to the patient morphology. When comparing 2 devices, this tissue thickness-induced variability in BC assessment might be even more complex, as the devices differ in terms of their X-ray spectra, X-ray filters, and differential attenuation equations. This same thickness-dependent variation could also explain the difference between the thick (head, trunk, and pelvis) and thin (arms and legs) body regions.

To date, 18 other studies have compared devices for which 16 provided calibration equations, but most did not perform multivariate analysis.^18–35^ These previous regression equations could not be validated in the OsteoLaus cohort because of the divergence between their participant characteristics and their devices compared with those from the OsteoLaus cohort. It remains unclear to what extent these standardization equations are generalizable, as none of the previously published equations, nor ours, have been externally validated. A careful analysis of the population and the devices (hard and software) is therefore essential before applying a calibration equation.

### Clinical impact of devices differences and the importance of standardization

In this study, we showed how BC-derived health conditions, such as sarcopenia, lipedema, cardiovascular risk, and obesity, were affected by device differences and were addressed using standardization. In the literature, no device-specific cut-offs have been reported for any diagnosis using BC assessment from whole-body DXA.^9^^,^^11^^,^^14^ Bennett et al. studied how assessing VAT with Lunar Prodigy and Hologic Discovery/A in 114 Chinese individuals affected cardiovascular risk classification.^19^ As in our study, the authors highlighted how the assessment of different devices directly affects the classification of BC-derived health conditions and how such differences became nonsignificant with the use of calibration equations. However, it remains unclear which device should be considered the gold standard, and which provides better discrimination regarding outcomes. To address these differences, either the BC parameters should be standardized to the same device used for diagnostic cut-off determination or the diagnostic cut-offs themselves should be device-specific. Both solutions lead to more accurate and precise diagnoses. For example, in osteoporosis management, the cut-offs are based on specific DXA regions, devices, and population references, and the DXA measures are first standardized and then compared to a cut-off.^47^ These considerations are particularly important and challenging when patients are followed up over long periods and might cross different health professionals and different devices. Better inter-device reliability would result in more accurate and reproducible measures and diagnostics.

### Strengths and limitations

The main strength of this study is that the OsteoLaus cohort is a large, representative, and modern cohort with up-to-date data sources and devices. The recency of a cohort minimizes ecological bias, which often cannot be considered due to the complexity of interactions. The large sample size allowed for more rigorous statistical analysis, including subgroup analyses and internal validation. We also used a Deming regression, which accounts for measurement error in both the explanatory and response variables. As such, both devices are set on the same level, and our hypothesis is gold standard free. However, without gold standard or clinical outcome, we cannot argue about which device performs best. Another strength is that all scans were taken within 1-h interval. This is important because daily variations in hydration status are known from previous studies (eg, water, caffeine, meals, medication, recent exercise, and medical conditions), and BC determined by DXA assumes a constant water fraction.^48^ Finally, the actual default setting from Lunar iDXA software detects and erases artifacts, whereas Hologic Horizon software considers artifacts as bone content and overestimates BMC. All DXA artifacts were preliminarily excluded or replaced on both devices, except for the hand rings, as the tissue differences were not significant.

This study is not without limitations. The first limitation arises from the hardware (different X-ray energies, filters, angles, and scan paths) and software (image reconstruction, tissue estimation, and calibration settings) of the device. These limitations support the rationale of this study and should encourage the use of standardization procedures. The most reported challenges by the technicians were the definition of the region of interest and the patients’ medical conditions, such as pain, reduced mobility, tremor, and inability to maintain the DXA position. To optimize positioning and minimize movements, radio-transparent cushions and sheets were available to help participants remain steady. In contrast to the ISCD guidelines, the patients’ hands were positioned on their side on H-Horizon-A due to its narrower exam table. Even though the projection of the hands is expected to have less area positioned on its side, the arms area was still greater in H-Horizon-A. This difference could explain the lower correlation and equation metrics observed for the arms. Participants with a BMI below 15 kg/m^2^ and above 37 kg/m^2^ were excluded during the OsteoLaus baseline visit, limiting the interpretability over these BMI ranges. Another limitation is that the OsteoLaus study included only women, and sex differences can affect weight and tissue distribution.^49^ Similarly, the majority of previous studies included younger participants, and only a few analyzed sex differences.^18^^,^^21^^,^^22^^,^^26–28^^,^^30^^,^^31^^,^^33^^,^^34^ As the OsteoLaus study is composed of 98.4% European participants, the results are not generalizable to Asian, African, and other populations.

### Conclusion

This comprehensive study, based on a large cohort of postmenopausal women, rigorously compared the L-iDXA and H-Horizon-A devices in the BC parameters assessment. Overall, BC parameters were highly correlated between devices. However, the differences between the devices for each BC parameter significantly and inconsistently varied based on the tissue type (lean, bone, fat) and body region. These differences were mainly attributed to hardware and software differences between the 2 available devices. The development of calibration equations for each BC parameter assessed by each device provides a good and internally validated approach to standardize these BC parameters. As the diagnostic cutoffs used in clinical settings are identical for both devices, the BC parameters measured from each device lead to different prevalences of BC-derived health conditions. These differences were addressed by the calibration equations applied to the validation dataset, improving the inter-device consistency for clinical practice. Further external validation in different populations is needed to assess the generalizability of the current standardization process. Given these DXA assessment’s variation and the importance of BC in the investigation and management of various health conditions, we encourage a systematic report of the DXA device and software used, the acquisition procedure, and the eventual use of a standardization technique in future studies and in clinical settings.

## Supplementary Material

SUPP_DATA_DXA_COMP_240617_R1_ziae088

## Data Availability

Some or all datasets generated during and/or analyzed during the current study are not publicly available but are available from the corresponding author upon reasonable request.
