# Supplementary material for: Standardization of body composition parameters between GE Lunar iDXA and Hologic Horizon A and their clinical impact
Source: JBMR Plus. 2024 Jul 10;8(9):ziae088. doi: 10.1093/jbmrpl/ziae088 (PMC11299512; doi:10.1093/jbmrpl/ziae088)
Supplement: SUPP_DATA_DXA_COMP_240617_R1_ziae088 [file supp_data_dxa_comp_240617_r1_ziae088.pdf]

Table S1.

TRIPOD Checklist: Prediction Model Development and Validation

Section/Topic	Item		Checklist Item	Page
**Title and abstract**				
Title	1	D;V	Identify the study as developing and/or validating a multivariable prediction model, the target population, and the outcome to be predicted.	0
Abstract	2	D;V	Provide a summary of objectives, study design, setting, participants, sample size, predictors, outcome, statistical analysis, results, and conclusions.	1
**Introduction**				
Background and objectives	3a	D;V	Explain the medical context (including whether diagnostic or prognostic) and rationale for developing or validating the multivariable prediction model, including references to existing models.	3
	3b	D;V	Specify the objectives, including whether the study describes the development or validation of the model or both.	3
**Methods**				
Source of data	4a	D;V	Describe the study design or source of data (e.g., randomized trial, cohort, or registry data), separately for the development and validation data sets, if applicable.	4
	4b	D;V	Specify the key study dates, including start of accrual; end of accrual; and, if applicable, end of follow-up.	4
Participants	5a	D;V	Specify key elements of the study setting (e.g., primary care, secondary care, general population) including number and location of centres.	4
	5b	D;V	Describe eligibility criteria for participants.	4 + Figure
	5c	D;V	Give details of treatments received, if relevant.	N/A
Outcome	6a	D;V	Clearly define the outcome that is predicted by the prediction model, including how and when assessed.	4-7
	6b	D;V	Report any actions to blind assessment of the outcome to be predicted.	N/A
Predictors	7a	D;V	Clearly define all predictors used in developing or validating the multivariable prediction model, including how and when they were measured.	4-7
	7b	D;V	Report any actions to blind assessment of predictors for the outcome and other predictors.	N/A
Sample size	8	D;V	Explain how the study size was arrived at.	4,6,9 + Figure
Missing data	9	D;V	Describe how missing data were handled (e.g., complete-case analysis, single imputation, multiple imputation) with details of any imputation method.	5-6, 9 + Figure
Statistical analysis methods	10a	D	Describe how predictors were handled in the analyses.	7-8
	10b	D	Specify type of model, all model-building procedures (including any predictor selection), and method for internal validation.	7-8
	10c	V	For validation, describe how the predictions were calculated.	8
	10d	D;V	Specify all measures used to assess model performance and, if relevant, to compare multiple models.	8
	10e	V	Describe any model updating (e.g., recalibration) arising from the validation, if done.	8
Risk groups	11	D;V	Provide details on how risk groups were created, if done.	8
Development vs. validation	12	V	For validation, identify any differences from the development data in setting, eligibility criteria, outcome, and predictors.	8
**Results**				
Participants	13a	D;V	Describe the flow of participants through the study, including the number of participants with and without the outcome and, if applicable, a summary of the follow-up time. A diagram may be helpful.	9 + Figure
	13b	D;V	Describe the characteristics of the participants (basic demographics, clinical features, available predictors), including the number of participants with missing data for predictors and outcome.	9 + Table
	13c	V	For validation, show a comparison with the development data of the distribution of important variables (demographics, predictors and outcome).	9 + Table
Model development	14a	D	Specify the number of participants and outcome events in each analysis.	9-10 + Tables
	14b	D	If done, report the unadjusted association between each candidate predictor and outcome.	10-11 + Tables
Model specification	15a	D	Present the full prediction model to allow predictions for individuals (i.e., all regression coefficients, and model intercept or baseline survival at a given time point).	10-11 + Tables
	15b	D	Explain how to the use the prediction model.	11
Model performance	16	D;V	Report performance measures (with CIs) for the prediction model.	10-11 + Tables
Model-updating	17	V	If done, report the results from any model updating (i.e., model specification, model performance).	Tables
**Discussion**				
Limitations	18	D;V	Discuss any limitations of the study (such as nonrepresentative sample, few events per predictor, missing data).	15-16
Interpretation	19a	V	For validation, discuss the results with reference to performance in the development data, and any other validation data.	13 + Tables
	19b	D;V	Give an overall interpretation of the results, considering objectives, limitations, results from similar studies, and other relevant evidence.	12-14
Implications	20	D;V	Discuss the potential clinical use of the model and implications for future research.	14-15
**Other information**				
Supplementary information	21	D;V	Provide information about the availability of supplementary resources, such as study protocol, Web calculator, and data sets.	17
Funding	22	D;V	Give the source of funding and the role of the funders for the present study.	17

*Items relevant only to the development of a prediction model are denoted by D, items relating solely to a validation of a prediction model are denoted by V, and items relating to both are denoted D;V. We recommend using the TRIPOD Checklist in conjunction with the TRIPOD Explanation and Elaboration document.

Figure S1. Regions and number of artifacts

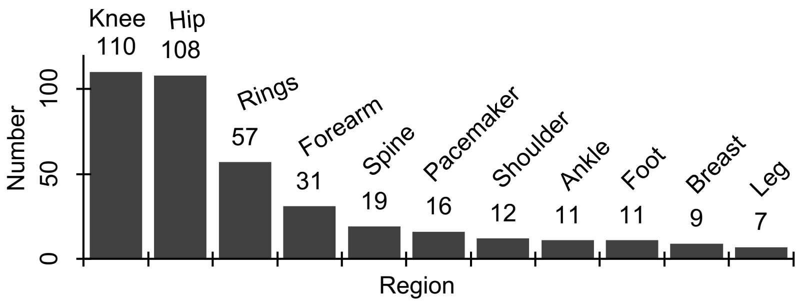

Legend: 308 participants out of 944 had 391 artifacts: All artifacts impacting regional and total body measures were excluded when image mirroring was not available.

<u>Figure S2.</u> (A) Bland-Altman analysis: Fat mass

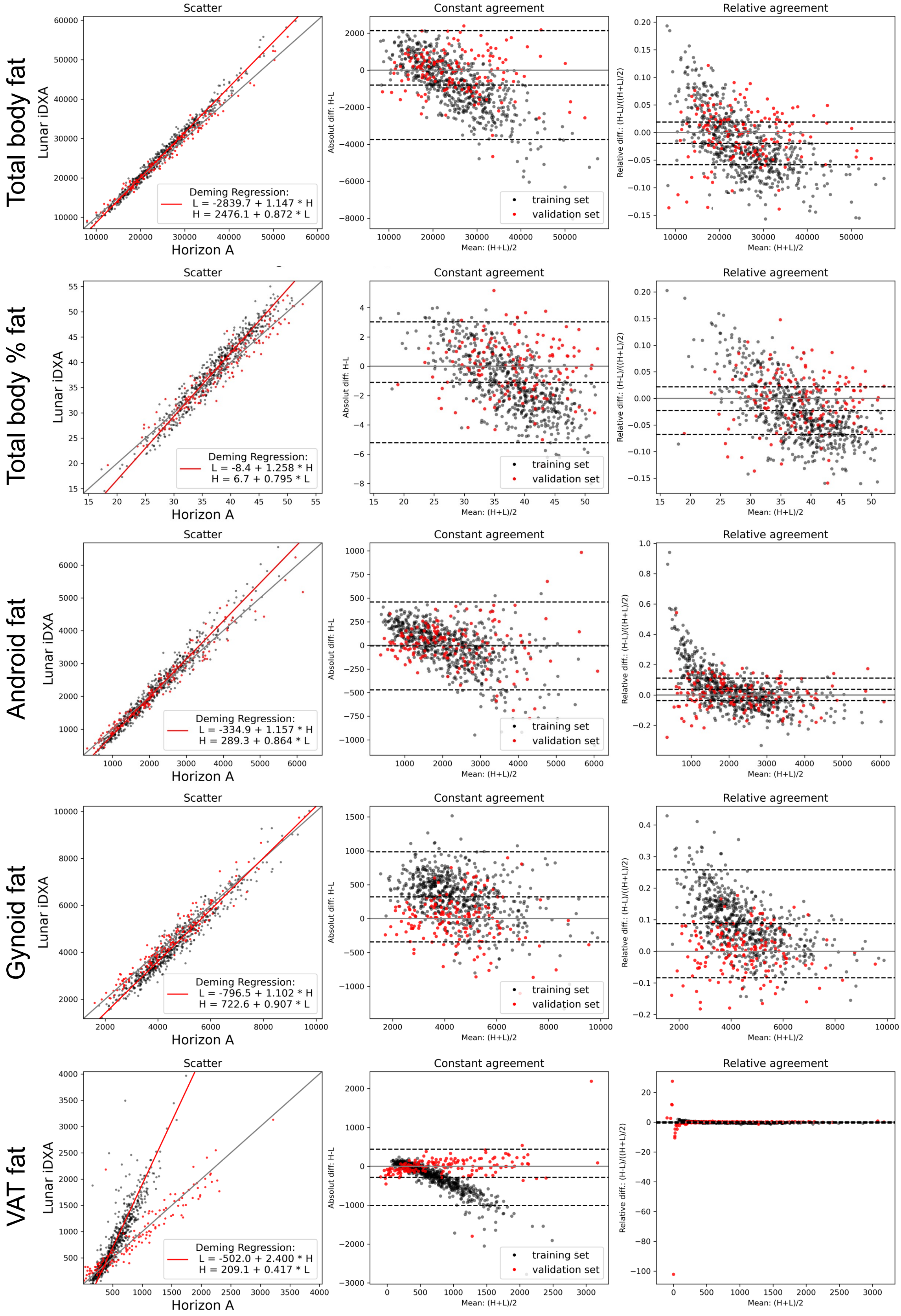

Symbols: — : identity line (in case of perfect match); — : regression line from test set; – – : mean ± 1.96 standard deviation from test set; ●: train set; ● : test set after calibration.

Legend: Bland-Altman describing the agreement of the paired values (X: Hologic Horizon A and Y: Lunar iDXA) including 3 graphs (see methodology); VAT: visceral fat.

Table S2. (A) Measures of fat mass: Univariate and multivariate model and evaluation metrics

		Univariate model■	$R^{2\ Train}$<br>$R^{2\ Test}$	MAE ^{Train}<br>MAE ^{Test}	Delta ^{Test}	p*	Multivariate model▲	$R^{2\ Train}$<br>$R^{2\ Test}$	MAE ^{Train}<br>MAE ^{Test}	Delta $^{c\ Test}$<br>(μ ± σ)	p*
Fat mass [gr.]	Total body	H= 2476.1 + 0.872*L_WBTOT_FAT<br>L= - 2839.7 + 1.147*H_WBTOT_FAT	0.986<br>0.983	718.29<br>792.63	-98.97<br>±1017.41	0.96	H: no multivariate model<br>L= - 3489.1 + 1.095*H_WBTOT_FAT<br>+ 0.124*H_WBTOT_LEAN<br>- 1.612*H_WBTOT_BMC	L:<br>0.989<br>0.987	L:<br>809.13<br>749.04	L:<br>109.15<br>±1029.51	L:<br>0.98
	Total body percent [%]	H= 6.71 + 0.795*L_WBTOT_PFAT<br>L= - 8.44 + 1.258*H_WBTOT_PFAT	0.942<br>0.921	1.10<br>1.22	-0.08<br>±1.53	0.95	H: no multivariate model<br>L= - 9.54 + 1.153*H_WBTOT_PFAT<br>+ 2.196e-04*H_WBTOT_LEAN<br>- 1.993e-03*H_WBTOT_BMC	L:<br>0.960<br>0.952	L:<br>1.21<br>1.16	L:<br>0.11<br>±1.53	L:<br>0.90
	Subtotal body	H= 2188.7 + 0.872*L_SUBTOT_FAT<br>L= - 2509.6 + 1.147*H_SUBTOT_FAT	0.986<br>0.982	724.17<br>808.38	-84.21<br>±1043.51	0.97	H: no multivariate model<br>L= - 3454.1 + 1.093*H_SUBTOT_FAT<br>+ 0.141*H_SUBTOT_LEAN<br>-2.124*H_SUBTOT_BMC	L:<br>0.989<br>0.987	L:<br>810.60<br>750.97	L:<br>95.21<br>±1032.14	L:<br>0.99
	Arms	H= 72.0 + 1.058*L_ARMS_FAT<br>L= - 68.0 + 0.945*H_ARMS_FAT	0.907<br>0.921	242.08<br>224.71	-16.69<br>±316.78	0.89	H: no multivariate model<br>L= - 127.6 + 0.889*H_ARMS_FAT<br>+ 0.132*H_ARMS_LEAN<br>- 1.078*H_ARMS_BMC	L:<br>0.913<br>0.925	L:<br>201.06<br>219.23	L:<br>10.27<br>±287.98	L:<br>0.98
	Legs	H= 706.5 + 0.975*L_LEGS_FAT<br>L= - 724.8 + 1.026*H_LEGS_FAT	0.977<br>0.979	341.89<br>313.37	-29.13<br>±399.38	0.98	H= 506.7 + 0.959*L_LEGS_FAT<br>+ 0.452*L_LEGS_BMC<br>L= - 460.1 + 1.005*H_LEGS_FAT<br>+ 0.075*H_LEGS_LEAN<br>- 1.446*H_LEGS_BMC	0.977<br>0.979	313.71<br>340.43	-24.35<br>±400.05	0.98
	Trunk	H= 846.8 + 0.810*L_TRUNK_FAT<br>L= - 1044.9 + 1.234*H_TRUNK_FAT	0.973<br>0.975	548.39<br>549.03	-49.25<br>±741.19	0.96	H= 508.1 + 0.806*L_TRUNK_FAT<br>+ 0.259*L_TRUNK_LEAN<br>- 1.128*L_TRUNK_BMC<br>L= - 1866.6 + 1.159*H_TRUNK_FAT<br>+ 0.165*H_TRUNK_LEAN<br>- 3.436*H_TRUNK_BMC	0.974<br>0.976	534.36<br>536.57	-45.68<br>±723.56	0.94
	Android	H= 289.3 + 0.864*L_ANDROID_FAT<br>L= - 334.9 + 1.157*H_ANDROID_FAT	0.964<br>0.962	130.14<br>142.12	6.43<br>±200.64	0.92	H: no multivariate model<br>L= - 485.4 + 1.094*H_ANDROID_FAT<br>+ 0.021*H_TRUNK_LEAN<br>- 0.303*H_TRUNK_BMC	L:<br>0.965<br>0.965	L:<br>163.59<br>149.22	L:<br>-4.13<br>±225.96	L:<br>0.89
	Gynoid	H= 744.8 + 0.901*L_GYNOID_FAT<br>L= - 827.0 + 1.110*H_GYNOID_FAT	0.950<br>0.939	219.99<br>227.61	8.77<br>±301.69	0.84	H= 559.2 + 0.870*L_GYNOID_FAT<br>+ 0.420*L_LEGS_BMC<br>L= - 632.7 + 1.062*H_GYNOID_FAT<br>+ 0.045*H_LEGS_LEAN<br>- 0.716*H_LEGS_BMC	0.952<br>0.942	219.13<br>217.41	13.57<br>±294.9	0.82
	VAT	H= 209.1 + 0.417*L_VAT<br>L= - 502.0 + 2.400*H_VAT	0.832<br>0.852	70.33<br>66.40	1.29<br>±118.5	089	H-L: no multivariate model.	-	-	-	-

Legend: ■Deming regression: linear regression using orthogonal distance regression; the evaluation metrics (R2, MAE and Delta) represent only the evaluation from L to H, and the reverse when L is mentioned; R^2: R-squared = proportion of variance explained by the model; MAE: Mean Absolute Error; Delta: difference between devices after calibration (mean +/- standard deviation); *p-value from Mann-Whitney U test; ▲Backward stepwise multivariate linear regression: if only one variable was left, no multivariate model was shown. *Abbreviations*: H: H-Horizon-A device A$^{\text{Classic setting}}$; L: L-iDXA device; WBTOT: total body; SUBTOT: Subtotal body; BMC: Bone Mineral Content; PFAT: percent fat = total fat mass / total mass; VAT: visceral adipose tissue.

Figure S2. (B) Bland-Altman analysis: Lean mass

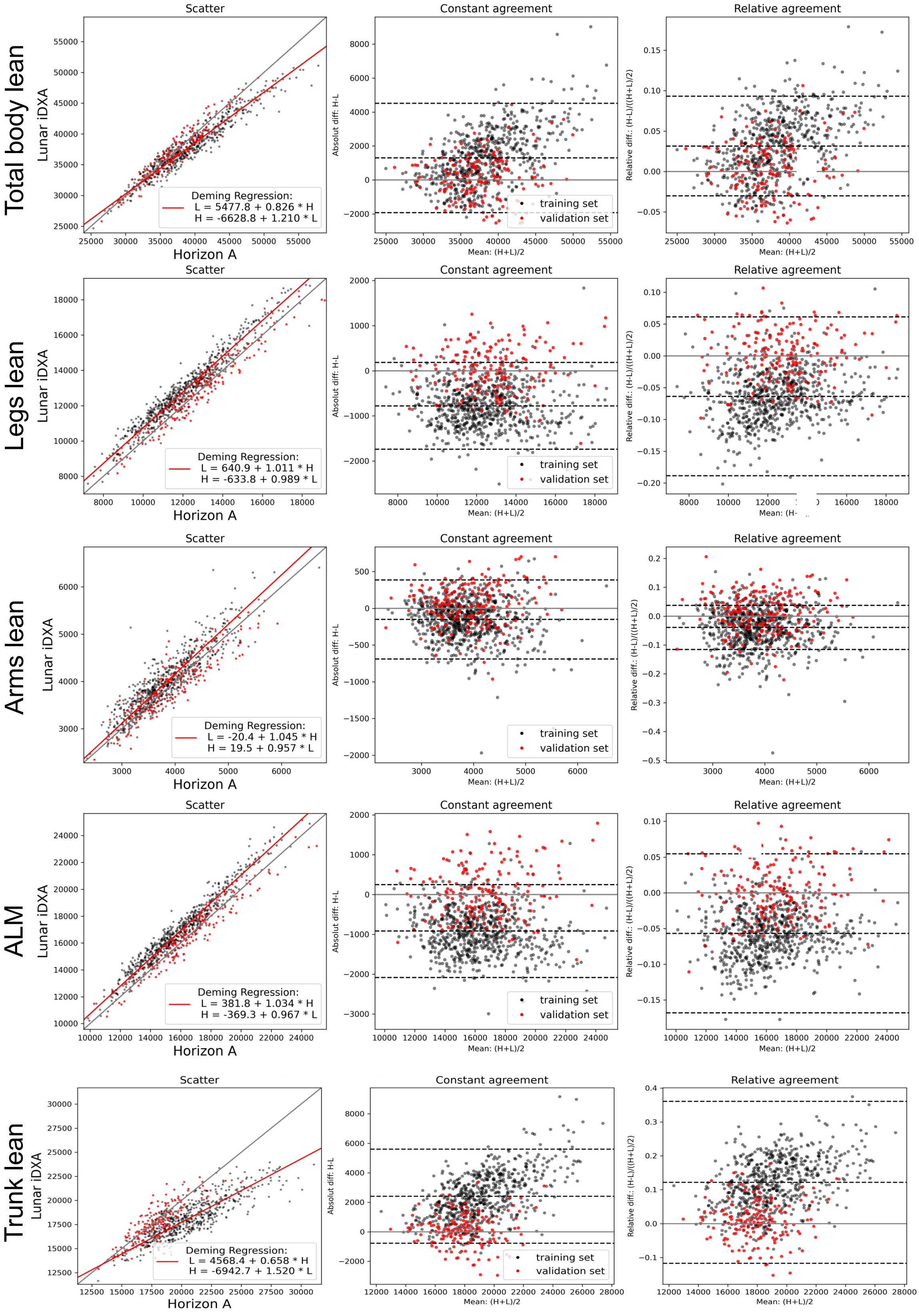

Symbols: — : identity line (in case of perfect match); — : regression line from test set; – – : mean ± 1.96 standard deviation from test set; ●: train set; ● : test set after calibration.
Legend: Bland-Altman describing the agreement of the paired values (X: Hologic Horizon A and Y: Lunar iDXA) including 3 graphs (see methodology); ALM: Appendicular Lean Mass.

Table S2. (B) Measures of lean mass: Univariate and multivariate model and evaluation metrics

		Univariate model■	R^2 Train<br>R^2 Test	MAE Train<br>MAE Test	Delta Test	p*	Multivariate model▲	R^2 Train<br>R^2 Test	MAE Train<br>MAE Test	Delta c Test<br>(μ ± σ)	p*
Lean mass [gr.]	Total body	H= - 6628.8 + 1.210*L_WBTOT_LEAN<br>L= 5477.8 + 0.826*H_WBTOT_LEAN	0.920<br>0.912	1197.61<br>1276.73	161.86<br>±1622.34	0.90	H= - 1885.9 + 0.978*L_WBTOT_LEAN + 0.156*L_WBTOT_FAT<br>L= 3882.5 + 0.883*H_WBTOT_LEAN - 0.114*H_WBTOT_FAT + 1.213*H_WBTOT_BMC	0.969<br>0.964	808.19<br>743.70	84.29<br>±1039.69	0.95
	Sub-total body	H= - 5882.1 + 1.212*L_SUBTOT_LEAN<br>L= 4854.9 + 0.825*H_SUBTOT_LEAN	0.915<br>0.908	1197.60<br>1256.38	193.47<br>±1609.37	0.90	H= - 1669.0 + 0.954*L_SUBTOT_LEAN + 0.155*L_SUBTOT_FAT + 0.556*L_SUBTOT_BMC<br>L= 3565.5 + 0.874*H_SUBTOT_LEAN - 0.115*H_SUBTOT_FAT + 1.660*H_SUBTOT_BMC	0.967<br>0.963	820.63<br>741.61	116.76<br>±1028.65	0.91
	Arms	H= 19.8 + 0.957*L_ARMS_LEAN<br>L= - 20.6 + 1.045*H_ARMS_LEAN	0.806<br>0.818	204.57<br>219.06	41.52<br>±272.79	0.70	H= 242.4 + 0.965*L_ARMS_LEAN - 0.088*L_ARMS_FAT<br>L= 231.07 + 0.768*H_ARMS_LEAN + 0.109*H_ARMS_FAT + 1.822*H_ARMS_BMC	0.830<br>0.828	206.04<br>188.88	-40.53<br>±264.99	0.77
	Legs	H= - 633.8 + 0.989*L_LEGS_LEAN<br>L= 640.9 + 1.011*H_LEGS_LEAN	0.938<br>0.936	381.94<br>405.25	35.55<br>±505.18	0.91	H : no multivariate model<br>L= 808.6 + 0.903*H_LEGS_LEAN + 0.040*H_LEGS_FAT + 1.081*H_LEGS_BMC	L: 0.944<br>0.945	L: 373.07<br>361.49	L: -37.11<br>±464.09	L:<br>0.92
	Trunk	H= - 6942.7 + 1.520*L_TRUNK_LEAN<br>L= 4568.4 + 0.658*H_TRUNK_LEAN	0.672<br>0.690	1332.13<br>1284.48	169.93<br>±1689.61	0.74	H= - 418.1 + 0.957*L_TRUNK_LEAN + 0.273*L_TRUNK_FAT<br>L=3379.4 + 0.865*H_TRUNK_LEAN - 0.242*H_TRUNK_FAT - 0.436*L_TRUNK_BMC	0.940<br>0.934	581.17<br>570.37	27.83<br>±783.24	0.95
	ALM	H= -369.3 + 0.967*L_ALM<br>L= 381.8 + 1.034*H_ALM	0.943<br>0.939	452.43<br>497.70	61.95<br>±626.66	0.89	H= - 102.2 + 0.9756*L_ALM – 0.035*L_AFM<br>L= 654.3 + 0.904*H_ALM + 0.067*H_AFM + 1.030*H_ABM	0.946<br>0.940	491.04<br>440.46	68.16<br>±621.39	0.95

Legend: ■Deming regression: linear regression using orthogonal distance regression; the evaluation metrics (R2, MAE and Delta) represent only the evaluation from L to H, and the reverse when L is mentioned; R^2: R-squared = proportion of variance explained by the model; MAE: Mean Absolute Error; Delta: difference between devices after calibration (mean +/- standard deviation); *p-value from Mann-Whitney U test; ▲Backward stepwise multivariate linear regression: if only one variable was left, no multivariate model was shown. *Abbreviations*: H: H-Horizon-A device A Classic setting; L: L-iDXA device; WBTOT: total body; SUBTOT: Subtotal body; BMC: Bone Mineral Content; ALM: Appendicular Lean Mass; ABM: Appendicular Bone Mass (variable created for this correction); AFM: Appendicular Fat Mass (variable created for this correction).

Figure S2. (C) Bland-Altman analysis: bone mass

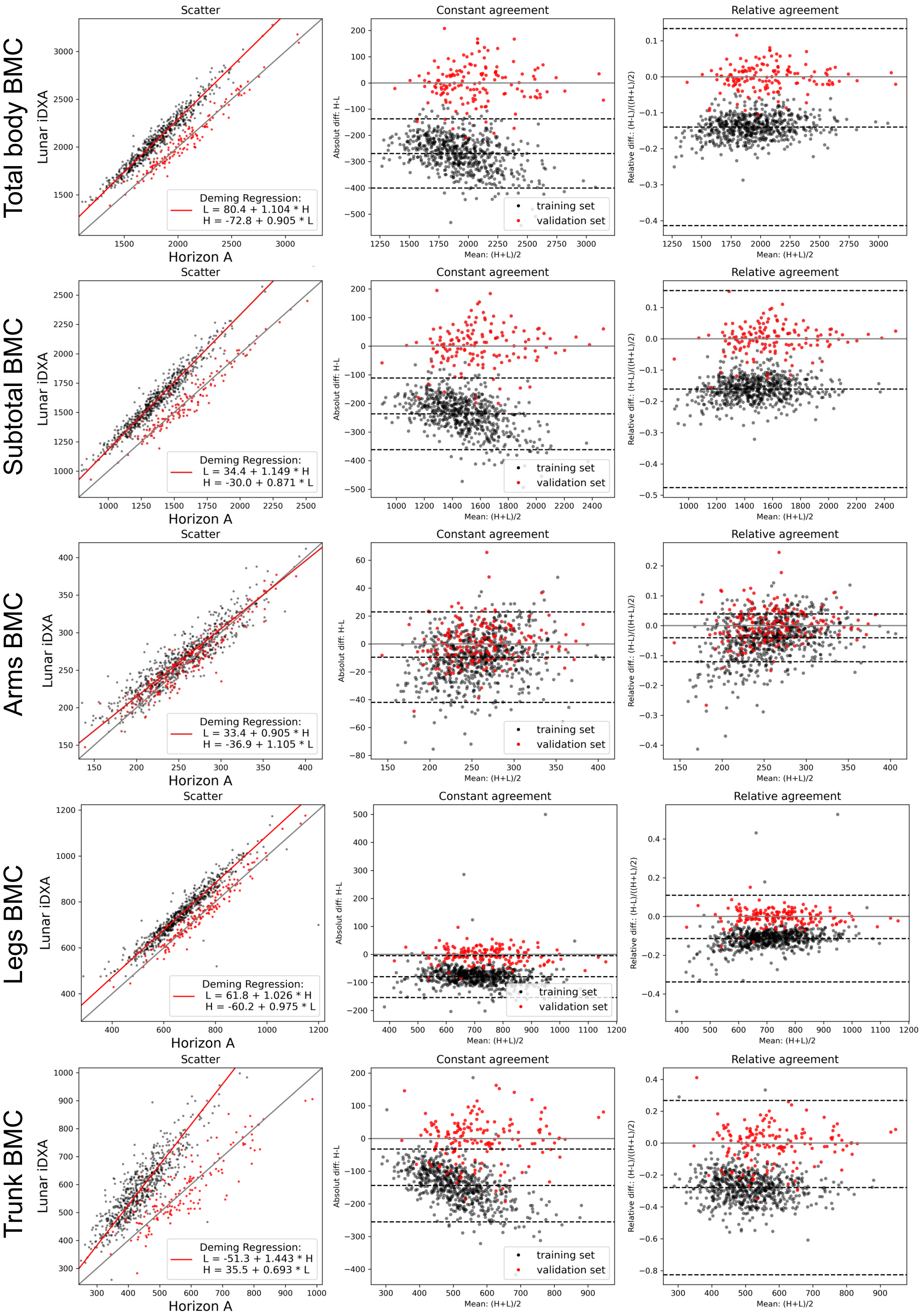

Symbols: — : identity line (in case of perfect match); — : regression line from test set; – – : mean ± 1.96 standard deviation from test set; ●: train set; ● : test set after calibration.

Legend: Bland-Altman describing the agreement of the paired values (X: Hologic Horizon A and Y: Lunar iDXA) including 3 graphs (see methodology); BMC: Bone Mineral Content

Table S2.1. (C) Bone mineral content: comparison of total body and regional measures, absolute and relative differences and proposed calibration equations

Tissue and region		n Train - Test	Train set Lunar	Hologic	Absolute difference	p*	Relative Difference	Corr-elation+	Equation▲	R^2 Train	R^2 Test
Bone mineral content [gr.]	Total body	620 - 146	2072.8 ±292.9	1803.9 ±265.8	-268.899 ±67.197	<0.001	-0.14 ±0.03	0.972	H= - 72.8 + 0.905*L_WBTOT_BMC<br>L= 80.4 + 1.104*H_WBTOT_BMC	H: 0.952<br>L: 0.951	0.947<br>0.946
	Subtotal body	620 - 146	1597.2 ±245.3	1360.7 ±214.4	-236.494 ±63.840	<0.001	-0.16 ±0.04	0.967	H= - 30.0 + 0.871*L_SUBTOT_BMC<br>L= 34.4 + 1.149*H_SUBTOT_BMC	H: 0.941<br>L: 0.940	0.932<br>0.929
	Subtotal density (BMD)	620 - 146	0.923 ±0.102	0.847 ±0.073	-0.076 ±0.041	<0.001	-0.08 ±0.04	0.940	H= 0.199 + 0.703*L_SUBTOT_BMD<br>L= - 0.283 + 1.423*H_SUBTOT_BMD	H: 0.889<br>L: 0.885	0.915<br>0.918
	Arms	734 - 181	261.6 ±39.5	252.1 ±43.4	-9.541 ±16.570	<0.001	-0.04 ±0.07	0.925	H= - 36.9 + 1.105*L_ARMS_BMC<br>L= 33.4 + 0.905*H_ARMS_BMC	H: 0.847<br>L: 0.849	0.895<br>0.885
	Legs	688 - 172	749.2 ±114.3	670.0 ±111.6	-79.174 ±38.171	<0.001	-0.11 ±0.06	0.961	H= - 60.2 + 0.975*L_LEGS_BMC<br>L= 61.8 + 1.026*H_LEGS_BMC	H: 0.887<br>L: 0.886	0.952<br>0.954
	Trunk	646 - 155	584.2 ±111.9	440.3 ±81.1	-143.916 ±56.904	<0.001	-0.28 ±0.09	0.863	H= 41.506 + 0.704*L_TRUNK_BMC<br>- 3.474e-03*L_TRUNK_FAT<br>+ 1.848e-03*L_TRUNK_LEAN<br>L= - 77.282 + 0.992*H_TRUNK_BMC<br>+ 2.326e-03*H_TRUNK_FAT<br>+ 9.705e-03*H_TRUNK_LEAN	H: 0.803<br>L: 0.848	0.790<br>0.853

Legend: n = number of participant finally included after exclusion and data splitting process (cf. fig. 1); Lunar (L), Hologic (H) and absolute difference (H-L) are expressed as mean +/- standard deviation in grams (gr.); *p-value from Mann-Whitney U test; Relative difference = H-L/((H+L)/2); +Spearman rank correlation coefficient; ▲Equations (see table A.2. C for the model selection): H: Hologic Horizon A[Classic setting]; L: Lunar iDXA; R^2: R-squared = proportion of variance explained by the model. *Abbreviations*: WBTOT: Whole Body Total; BMC: Bone Mineral Content; SUBTOT: Subtotal body; BMD: Bone Mineral Density.

Table S2.2. (C) Measures of bone mass: Univariate and multivariate model and evaluation metrics

		Univariate model■	$R^{2\ Train}$<br>$R^{2\ Test}$	MAE ^{Train}<br>MAE ^{Test}	Delta$^{c\ Test}$	p*	Multivariate model▲	$R^{2\ Train}$<br>$R^{2\ Test}$	MAE ^{Train}<br>MAE ^{Test}	Delta$^{c\ Test}$<br>(μ ± σ)	p*
Bone mass [gr.]	Total body	H= -72.8 +0.905*L_WBTOT_BMC<br>L= 80.4 + 1.104*H_WBTOT_BMC	0.952<br>0.947	45.86<br>50.06	-1.85<br>±65.68	0.99	H= 46.4 + 0.944*L_WBTOT_BMC<br>-1.591e-03*L_WBTOT_FAT<br>- 4.253e-03*L_WBTOT_LEAN<br>L= - 52.3 + 0.980*H_WBTOT_BMC<br>- 1.055e-03*H_WBTOT_FAT<br>+ 9.826e-03*H_WBTOT_LEAN	0.960<br>0.957	45.13<br>41.05	-2.07<br>±58.87	1.0
	Subtotal body	H= -30.0 +0.871*L_SUBTOT_BMC<br>L= 34.4 + 1.149*H_SUBTOT_BMC	0.941<br>0.932	40.59<br>45.39	0.87<br>±60.82	0.90	H= 41.8 + 0.892*L_SUBTOT_BMC<br>- 1.105e-03*L_SUBTOT_FAT<br>- 2.283e-03*L_SUBTOT_LEAN<br>L= - 54.1 + 0.994*H_SUBTOT_BMC<br>- 1.330e-03*H_SUBTOT_FAT<br>+ 9.114e-03*H_SUBTOT_LEAN	0.945<br>0.938	43.9<br>38.8	0.76<br>±58.17	0.93
	Sub-total density (BMD)	H= 0.199 +0.703*L_SUBTOT_BMD<br>L= -0.283+1.423*H_SUBTOT_BMD	0.889<br>0.915	0.02<br>0.02	0.0<br>±0.02	0.97	H= 0.2 + 0.714*L_SUBTOT_BMD<br>- 2.085e-06*L_SUBTOT_FAT<br>+ 1.338e-06*L_SUBTOT_LEAN<br>L= - 0.217 + 1.284*H_SUBTOT_BMD<br>+ 3.223e-06*H_SUBTOT_FAT<br>- 6.783e-07*H_SUBTOT_LEAN	0.935<br>0.945	0.01<br>0.01	0.0<br>±0.02	0.94
	Arms	H= -36.9 + 1.105*L_ARMS_BMC<br>L= 33.4 + 0.905*H_ARMS_BMC	0.847<br>0.895	13.07<br>11.99	1.05<br>±15.86	0.77	H= - 20.718 + 0.961*L_ARMS_BMC<br>+ 7.448e-03*L_ARMS_FAT<br>L= 40.0 + 0.887*H_ARMS_BMC<br>- 7.997e-03*H_ARMS_FAT<br>+ 5.954e-03*H_ARMS_LEAN	0.887<br>0.916	10.67<br>11.04	0.96<br>±14.18	0.82
	Legs	H= -60.2 + 0.975*L_LEGS_BMC<br>L= 61.8 + 1.026*H_LEGS_BMC	0.887<br>0.952	21.38<br>19.86	-1.88<br>±25.92	0.97	H= - 19.0 + 0.923*L_LEGS_BMC<br>+ 3.887e-03*L_LEGS_FAT<br>- 2.915e-03*L_LEGS_LEAN<br>L= 67.1 + 0.919*H_LEGS_BMC<br>- 5.312e-03*H_LEGS_FAT<br>+ 9.621e-03*H_LEGS_LEAN	0.897<br>0.961	17.63<br>19.78	-2.94<br>±23.14	0.92
	Trunk	H= 35.5 + 0.693*L_TRUNK_BMC<br>L= -51.3 + 1.443*H_TRUNK_BMC	0.757<br>0.712	30.11<br>33.86	-1.78<br>±45.05	0.71	H= 41.5 + 0.704*L_TRUNK_BMC<br>- 3.474e-03*L_TRUNK_FAT<br>+ 1.848e-03*L_TRUNK_LEAN<br>L= - 77.3 + 0.992*H_TRUNK_BMC<br>+ 2.326e-03*H_TRUNK_FAT<br>+ 9.705e-03*H_TRUNK_LEAN	0.803<br>0.790	28.97<br>26.76	-0.51<br>±38.47	0.84

Legend: ■Deming regression: linear regression using orthogonal distance regression; the evaluation metrics (R2, MAE and Delta) represent only the evaluation from L to H, and the reverse when L is mentioned; R^2: R-squared = proportion of variance explained by the model; MAE: Mean Absolute Error; Delta: difference between devices after calibration (mean +/- standard deviation); *p-value from Mann-Whitney U test; ▲Backward stepwise multivariate linear regression: if only one variable was left, no multivariate model was shown.
Abbreviations: H: H-Horizon-A device A$^{Classic\ setting}$; L: L-iDXA device; WBTOT: total body; SUBTOT: Subtotal body; BMC: Bone Mineral Content; BMD: Bone Mineral Density.

Figure S3.1. Interactions between tissues for trunk bone mineral content (BMC) through models, covariates and subgroups visualization

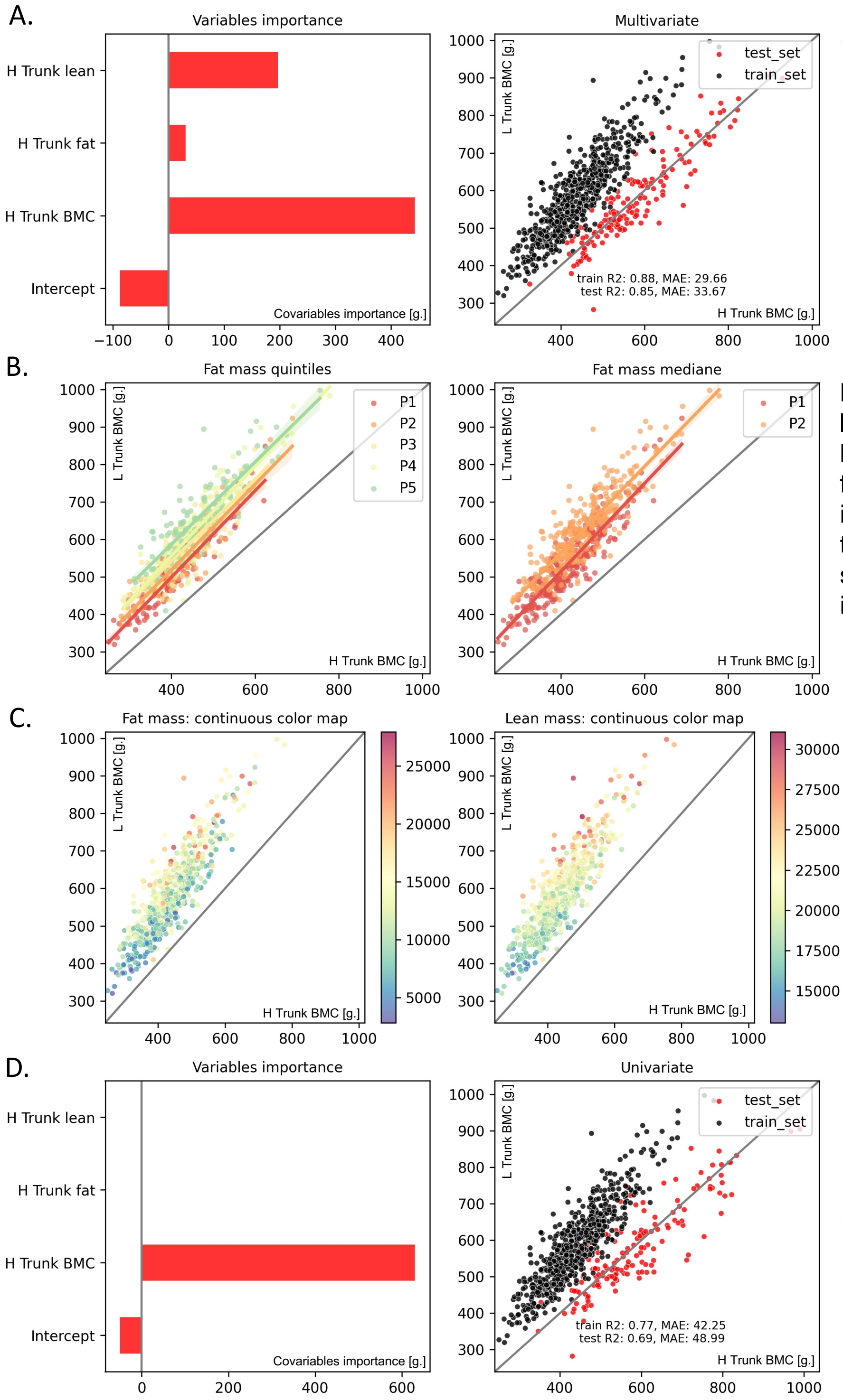

A. Multivariate model: Left) visualisation of the impact of each predicted covariables in the final predicted value. Each red bar correspond to the mean value of a covariables multiplied by its coefficient. Right) predicted values in the test set (red) based on the multivariate model.

B. Percentile sub-groups and linear regressions between both devices. In this example, fat mass seems to significantly impact the intercept, visible by the translation of the regre-ssion lines), but do not visually impact the slope of equations.

C. Continuous colors mapp-ing of fat and lean values [g.] and scatterplot of both dev-ices. Both lean and fat mass seems to increase the be-tween devices differences: the color gradient is slightly visible between the devices, perpendicularly to the line of identity (grey line).

D. Univariate model, see de-scription in A for the graphics. The multivariate model (A) results in higher R2 and Mean Absolute Error (MAE), in both train and test dataset, representing a better fit.

<u>Figure S3.2.</u> Interactions between tissues for appendicular lean (ALM) through models, covariates and subgroups visualization

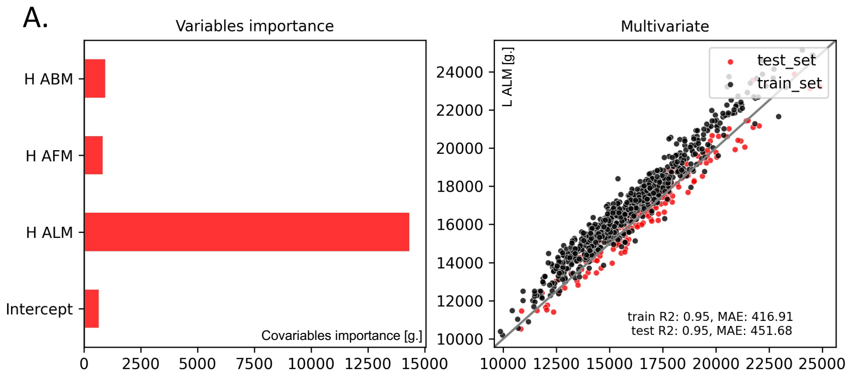

A. Multivariate model:
Left) visualisation of the impact of each predicted covariables in the final predicted value. Each red bar correspond to the mean value of a covariables multiplied by its coefficient.
Right) predicted values in the test set (red) based on the multivariate model.

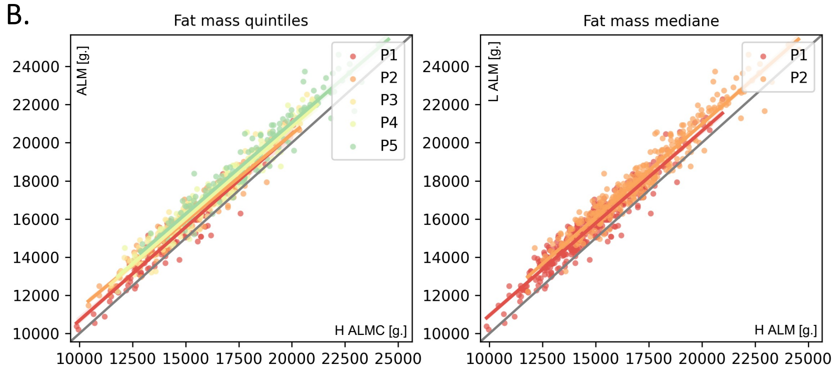

B. In this percentile subgroup analysis, fat mass changes only slightly impact the intercept but not the slope of the regression equation, as opposed to previous example of trunk BMC (Figure S3.1)

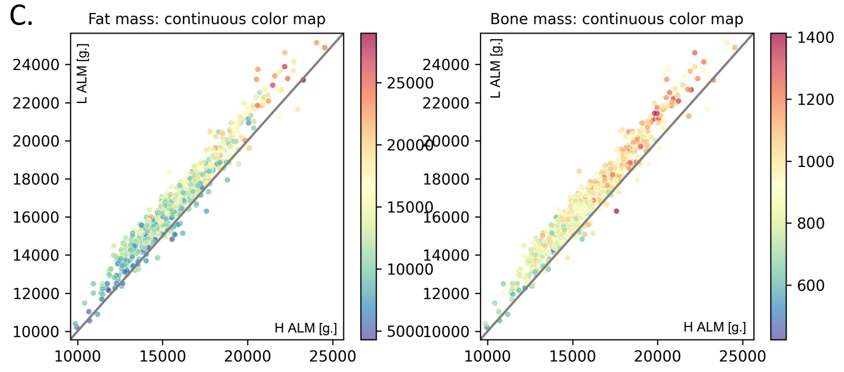

C. Continuous color mapping of fat and bone values [g.] and scatterplot of both devices. Limited interaction of lean with the range of appendicular fat (AFM) or bone (ABM) mass seems visible.

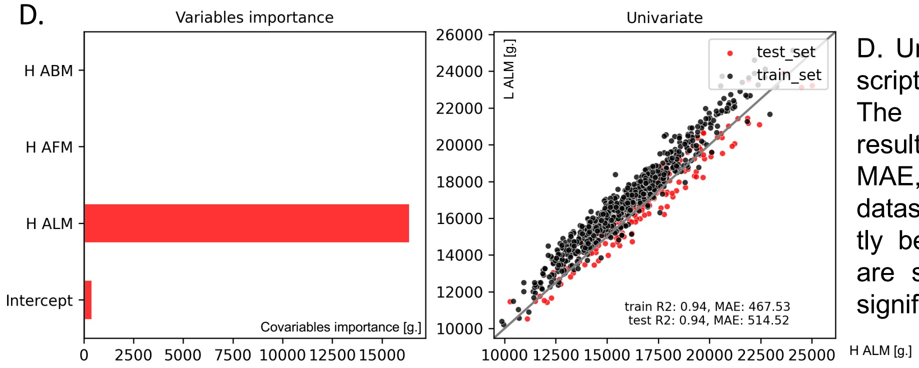

D. Univariate model, see description in A for the graphics. The multivariate model (A) result in slightly higher R2 and MAE, in both train and test dataset, representing a slightly better fit. The differences are small and the statistical significance limited.

Table S3. Absolute and relative differences between Lunar iDXA and Hologic Horizon A total body and regional measures of fat and lean mass using Hologic Classic vs. NHANES calibration setting

			Hologic	Lunar	Absolute diff.	*p-value	Relative diff.	Correlation coefficient[+]
Fat mass [gr.]	Total body	N	26906.3 ±7991.3	25602.8 ±8921.7	1303.5 ±1335.8	0.002	0.07 ±0.07	0.993
		C	24800.4 ±7786.6		-802.4 ±1501.2	0.19	-0.02 ±0.06	0.993
	Total body percent [%]	N	40.3 ±5.4	38.1 ±7.1	2.1 ±2.3	<0.001	0.06 ±0.07	0.969
		C	37.0 ±5.7		-1.1 ±2.1	0.001	-0.02 ±0.06	0.968
	Subtotal body	N	25722.0 ±7919.3	24742.7 ±8847.0	979.3 ±1338.3	0.02	0.06 ±0.07	0.993
		C	23766.8 ±7723.0		-975.9 ±1499.0	0.09	-0.03 ±0.06	0.993
	Arms	N	3283.1 ±1135.0	2870.7 ±1090.7	412.4 ±288.1	<0.001	0.15 ±0.09	0.969
		C	3110.4 ±1151.4		239.7 ±346.9	<0.001	0.08 ±0.11	0.955
	Legs	N	10001.7 ±3040.6	8867.9 ±3052.5	1133.8 ±461.7	<0.001	0.13 ±0.06	0.988
		C	9350.8 ±2976.4		482.9 ±463.1	0.001	0.06 ±0.06	0.987
	Trunk	N	12636.7 ±4537.5	13188.5 ±5440.0	-551.8 ±1202.5	0.10	-0.02 ±0.11	0.989
		C	11534.5 ±4420.8		-1654.06 ±1298.9	<0.001	-0.12 ±0.09	0.989
Lean mass [gr.]	Total body	N	36891.6 ±5066.9	37703.7 ±4459.4	-812.2 ±1470.9	<0.001	-0.02 ±0.04	0.953
		C	38997.5 ±5356.2		1293.7 ±1643.5	<0.001	0.03 ±0.04	0.953
	Subtotal body	N	34251.8 ±4906.2	34738.5 ±4315.0	-486.7 ±1463.4	0.01	-0.02 ±0.04	0.949
		C	36207.0 ±5186.2		1468.5 ±1628.9	<0.001	0.04 ±0.04	0.949
	Arrns	N	3604.4 ±575.5	3961.5 ±633.1	-357.2 ±272.3	<0.001	-0.09 ±0.07	0.898
		C	3810.1 ±608.3		-151.4 ±274.8	<0.001	-0.04 ±0.07	0.898
	Legs	N	11402.9 ±1849.9	12830.9 ±1977.0	-1428.0 ±492.8	<0.001	-0.12 ±0.04	0.966
		C	12053.8 ±1955.5		-777.1 ±490.0	<0.001	-0.06 ±0.04	0.966
	ALM	N	14997.4 ±2304.4	16770.7 ±2515.9	-1773.4 ±610.5	<0.001	-0.11 ±0.04	0.967
		C	15853.5 ±2436.0		-917.3 ±594.3	<0.001	-0.06 ±0.04	0.967
	Trunk	N	19310.0 ±2797.5	17999.6 ±2068.2	1310.4 ±1514.6	<0.001	0.07 ±0.08	0.822
		C	20412.3 ±2957.2		2412.6 ±1628.9	<0.001	0.12 ±0.08	0.822

Legend: Hologic (H, N:NHANES, C:Classic) , Lunar (L) and absolute difference (H-L) are expressed as mean +/- standard deviation in grams (gr.); *p-value from Mann-Whitney U test; Relative difference = H-L/((H+L)/2); [+]Spearman rank correlation coefficient.
Abbreviations: ALM: Appendicular Lean Mass; VFAT: Visceral fat mass.

Table S4. Absolute and relative differences between Lunar iDXA and Hologic Horizon A measures of a Whole-Body Hologic Phantom scanned 10 times

		Lunar	Hologic	Absolute diff.	*p-value	Relative diff.
Fat mass [gr.]	Total body	14676.9 ±55.5	14366.2 ±71.8	-310.7 ±101.9	<0.001	-0.02 ±0.01
	Arms	2627.0 ±17.6	1926.9 ±76.6	-700.0 ±83.8	<0.001	-0.31 ±0.04
	Legs	2151.0 ±29.4	2394.8 ±75.7	243.7 ±89.0	<0.001	0.11 ±0.04
	Trunk	8771.9 ±34.9	8538.6 ±81.0	-233.3 ±97.3	<0.001	-0.03 ±0.01
	VAT	832.1 ±221.7	1204.7 ±16.8	372.7 ±228.2	<0.001	0.39 ±0.27
Lean mass [gr.]	Total body	13153.0 ±61.1	15121.6 ±62.6	1968.5 ±94.2	<0.001	0.14 ±0.01
	Arms	2135.8 ±15.5	2272.3 ±52.4	136.5 ±51.9	<0.001	0.06 ±0.02
	Legs	3420.3 ±32.4	3490.8 ±55.1	70.5 ±58.1	0.002	0.02 ±0.02
	Trunk	4348.1 ±54.6	5732.8 ±102.4	1384.6 ±111.0	<0.001	0.28 ±0.02
	ALM	5556.1 ±36.1	5763.1 ±80.6	207.0 ±70.6	<0.001	0.04 ±0.01
Bone mass [gr.]	Total body	672.8 ±3.9	709.6 ±9.8	36.8 ±7.7	<0.001	0.05 ±0.01
	Arms	56.5 ±0.8	50.0 ±2.6	-6.5 ±2.4	<0.001	-0.12 ±0.05
	Legs	230.3 ±1.4	238.4 ±2.7	8.1 ±3.7	<0.001	0.04 ±0.02
	Trunk	218.2 ±2.2	234.6 ±2.8	16.4 ±2.2	<0.001	0.07 ±0.01
Total mass [gr.]	Total body	28502.7 ±4.9	29487.7 ±59.4	985.0 ±59.3	<0.001	0.03 ±0.002
	Arms	4819.2 ±10.0	4249.2 ±121.5	-570.0 ±121.0	<0.001	-0.13 ±0.03
	Legs	5801.6 ±4.1	6123.9 ±94.9	322.4 ±93.9	<0.001	0.05 ±0.02
	Trunk	13338.3 ±23.4	14271.4 ±142.5	933.1 ±145.1	<0.001	0.07 ±0.01
Area [cm^2]	Total body	609.0 ±15.2	646.1 ±5.2	37.1 ±14.9	<0.001	0.06 ±0.02
	Arms	83.6 ±2.4	77.0 ±2.4	-6.6 ±3.9	<0.001	-0.08 ±0.05
	Legs	194.2 ±4.6	188.3 ±2.2	-5.8 ±5.4	0.005	-0.03 ±0.03
	Trunk	227.3 ±9.6	269.2 ±4.8	41.9 ±11.0	<0.001	0.17 ±0.05

Legend: BMC: Bone Mineral Content; ALM: Appendicular Lean Mass; VFAT: Visceral fat mass; *p-value from Mann-Whitney U test
